# Role of *MicroRNAs-221/222* in Digestive Systems

**DOI:** 10.3390/jcm4081566

**Published:** 2015-08-06

**Authors:** Juntaro Matsuzaki, Hidekazu Suzuki

**Affiliations:** 1 Center for Preventive Medicine, Keio University Hospital, Tokyo 160-0016, Japan; E-Mail: juntaro.matsuzaki@gmail.com; 2 Division of Gastroenterology and Hepatology, Department of Internal Medicine, Keio University School of Medicine, 35 Shinanomachi, Shinjuku-ku, Tokyo 160-8582, Japan

**Keywords:** microRNA, colorectal cancer, hepatocellular carcinoma, pancreatic cancer

## Abstract

*MiR-221* and *miR-222* (*miR-221/222*) are well-studied oncogenic microRNAs that are frequently upregulated in several types of human tumors, such as esophageal adenocarcinoma, gastric adenocarcinoma, colorectal adenocarcinoma, hepatocellular carcinoma, and pancreatic ductal adenocarcinoma. In these cancers, silencing *miR-221/222* could represent a novel anti-tumor approach to inhibit tumor growth and metastasis. On the other hand, *miR-221/222* also play onco-suppressive roles in cholangiocarcinoma and gastrointestinal stromal tumors (GISTs). Here we will review the roles of *miR-221/222* in digestive systems and their possibility as prognostic and therapeutic tools.

## 1. Introduction

MicroRNAs (miRs) are ~22 nucleotide noncoding RNAs that can downregulate various gene products by translational repression when partially complementary sequences are present in the 3′ untranslated regions (3′ UTR) of the target mRNAs or by directing mRNA degradation. Increasing evidence has demonstrated that miRs are involved in cancer initiation, progression, and metastasis, and may serve as diagnostic and prognostic biomarkers for cancers. Among the many miRNAs already identified as regulators of neoplastic transformation, invasion, and metastasis, *miR-221* and *miR-222* (*miR-221/222*) have emerged as key miRNAs deregulated in many cancers, such as gastrointestinal cancers, breast cancer, prostate cancer, thyroid cancer, and glioma [1,2,3,4,5]. *MiR-221* and *miR-222* are encoded in tandem from a gene cluster located on chromosome Xp11.3. Several reports indicated that *miR-221/222* could be used as a therapeutic tool to decrease cell proliferation or modulate sensitivity to anti-cancer agents [6,7,8]. Here we review the current knowledge about the role of *miR-221/222* in digestive systems, including hepatobiliary and pancreatic cancers.

## 2. Direct Targets of *miR-221/222*

The identification of target mRNAs is a key step for assessing the role of aberrantly expressed microRNAs in human cancer. To date, various direct targets of *miR-221/222* have been reported, even in the digestive system, as shown in Table 1. Among them, regulation of p27Kip1 by *miR-221/222* is well studied. Downregulation of p27Kip1 is required for cell cycle entry after growth factor stimulation. *MiR-221/222* are underactive towards p27Kip1-3′ UTRs in quiescent cells, as a result of target site hindrance. Pumilio-1 (PUM1) is a ubiquitously expressed RNA-binding protein (RBP) that interacts with p27Kip1-3′ UTR. In response to growth factor stimulation, PUM1 is upregulated and phosphorylated for optimal induction of its RNA-binding activity towards the p27Kip1-3′ UTR [9]. PUM1 binding induces a local change in RNA structure that favors association with *miR-221/222*, efficient suppression of p27Kip1 expression, and rapid entry to the cell cycle.

**Table 1:** Direct targets of *miR-221/222*.

Target	Cancer Type	Reference
p27Kip1	Esophageal adenocarcinoma	Matsuzaki *et al.* (2013)
	Hepatocellular carcinoma	Pineau *et al.* (2010), Fu *et al.* (2011), Callegari *et al.* (2012)
	Pancreatic ductal adenocarcinoma	Park *et al.* (2009), Sarkar *et al.* (2013), Tanaka *et al.* (2015)
p57Kip2	Colorectal adenocarcinoma	Sun *et al.* (2011)
	Pancreatic ductal adenocarcinoma	Sarkar *et al.* (2013)
PTEN	Gastric adenocarcinoma	Chun-Zhi *et al.* (2010)
	Hepatocellular carcinoma	Fornari *et al.* (2008), Callegari *et al.* (2012), Garofalo *et al.* (2009)
	Colorectal adenocarcinoma	Tsunoda *et al.* (2011), Xue *et al.* (2013)
	Pancreatic ductal adenocarcinoma	Sarkar *et al.* (2013)
RelA	Colorectal adenocarcinoma	Liu *et al.* (2014)
PDLIM2	Colorectal adenocarcinoma	Liu *et al.* (2014)
RECK	Colorectal adenocarcinoma	Qin *et al.* (2014)
BMF	Hepatocellular carcinoma	Gramantieri *et al.* (2009), Callegari *et al.* (2012), He *et al.* (2014)
BBC3	Hepatocellular carcinoma	He *et al.* (2014)
ANGPTL2	Hepatocellular carcinoma	He *et al.* (2014)
HDAC6	Hepatocellular carcinoma	Bae *et al.* (2015)
ERα	Hepatocellular carcinoma	Chen *et al.* (2015)
SOCS1	Hepatocellular carcinoma	Xu *et al.* (2014)
SOCS3	Hepatocellular carcinoma	Xu *et al.* (2014)
MDM2	Hepatocellular carcinoma	Fornari *et al.* (2014)
DDIT4	Hepatocellular carcinoma	Pineau *et al.* (2010)
TIMP3	Hepatocellular carcinoma	Garofalo *et al.* (2009)
TIMP2	Pancreatic ductal adenocarcinoma	Xu *et al.* (2015)
PIK3R1	Colangiocarcinoma	Okamoto *et al.* (2013)
PUMA	Pancreatic ductal adenocarcinoma	Sarkar *et al.* (2013)
TRPS1	Pancreatic ductal adenocarcinoma	Su *et al.* (2013)
KIT	Gastrointestinal stromal tumor	Koelz *et al.* (2011), Gits *et al.* (2013), Ihle *et al.* (2015)

## 3. Esophageal Cancer

Duodeno-gastro-esophageal bile reflux contributes to development of esophageal adenocarcinoma. We recently reported that expression levels of *miR-221/222* increased, along with the activity of nuclear bile acid receptor/farnesoid X receptor (FXR), when cultured esophageal epithelial cells were exposed to bile acids [10]. Furthermore, *miR-221/222* expression was higher in esophageal adenocarcinoma than in the surrounding Barrett’s esophagus, a precursor lesion of esophageal adenocarcinoma. p27Kip1 is known to inhibit the proteasomal protein degradation of the transcription factor CDX2. We also confirmed that the levels of p27Kip1 and CDX2 were lower in areas of esophageal adenocarcinoma than in those of Barrett’s esophagus. Incubation of cells with bile acids increased degradation of CDX2; this process was reduced when cells were also incubated with proteasome inhibitors. Overexpression of *miR-221/222* reduced levels of p27Kip1 and CDX2, and knockdown of *miR-221/222* increased levels of these proteins in cultured cells. In addition, inhibitors of *miR-221/222* reduced growth of xenograft tumors in immunodeficient mice.

## 4. Gastric Cancer

Liu *et al.* reported that *miR-221* was upregulated in 88% of gastric cancer tissue samples compared with their paired adjacent non-tumor tissue samples [11]. High expression of *miR-221* showed a significant correlation with advanced tumor-node-metastasis stage, local invasion, and lymphatic metastasis. *MiR-221* overexpression was an unfavorable prognostic factor for overall survival in patients with gastric cancer.

In gastric cancer cells, upregulation of *miR-221/222* induced the malignant phenotype, whereas knockdown of *miR-221/222* reversed this phenotype via induction of PTEN, a direct target of *miR-221/222* [12]. In addition, knockdown of *miR-221/222* inhibited cell growth and invasion and increased the radiosensitivity.

## 5. Colorectal Cancer

*MiR-221* was upregulated in 90% of colorectal cancer (CRC) tissue samples compared to that in the adjacent non-tumorous tissue, and the expression level was positively correlated to an advanced TNM stage and local invasion [13,14,15,16,17,18]. A survival analysis indicated that high expression of *miR-221* was closely associated with a shorter survival time [14,19]. In CRC cells, *miR-221* overexpression enhances, whereas *miR-221* depletion reduces CRC cell proliferation, migration, invasion, and colony formation [16,17]. In mice with colitis, injection of lentiviruses expressing *miR-221/222* sponges led to formation of fewer tumors than injection of control lentiviruses [16]. Protein expressions of p57Kip2 and RECK, direct targets of *miR-221*, were decreased in the CRC tissues, and promoted CRC occurrence and progress [15,17].

Liu *et al.* reported that mimics of *miR-221/222* activated NF-κB and STAT3 in CRC cells [16]. *MiR-221/222* also reduced the ubiquitination and degradation of the RelA and STAT3 proteins by binding to the 3′ untranslated region of PDLIM2 mRNA (PDLIM2 is a nuclear ubiquitin E3 ligase for RelA and STAT3). In human CRC tissues, levels of *miR-221/222* positively correlated with levels of RelA and STAT3 mRNAs. Levels of PDLIM2 mRNA were lower than non-tumor tissues.

Xue *et al.* investigated the regulative effect of *miR-221* on CRC cell radiosensitivity [20]. X-ray radiation had an effect on the expression of *miR-221* in CRC cells in a dose-dependent manner. The protein levels of PTEN, a direct target of *miR-221*, reduced gradually during exposure to X-rays. Inhibition of *miR-221* upregulated expression of PTEN protein and enhanced the radiosensitivity. Moreover, the inhibitory effect was dramatically abolished by pretreatment with anti-PTEN-siRNA, suggesting that the enhancement of radiosensitivity was mediated by PTEN.

Tsunoda *et al.* reported that the increased expression of *miR-221/222* was observed in 3D culture as compared with 2D culture [18]. They showed that *miR-221/222* was regulated by oncogenic KRAS, which plays several key roles in 3D culture. The protein expression level of PTEN was reduced under the control of KRAS in a 3D-specific manner.

The plasma concentration of *miR-221* is a potential biomarker for differentiating CRC patients from controls [21]. Kaplan–Meier curve assessment shows that the elevated plasma *miR-221* level is a significant prognostic factor for poor overall survival in CRC patients. The immunohistochemistry analysis demonstrates a significant correlation between plasma *miR-221* level and p53 expression.

Stool-based *miR-221* can also be used as a non-invasive biomarker for the detection of CRC [13]. In stool samples, *miR-221* showed a significant increasing trend from normal controls to late stages of CRC. The AUC of stool *miR-221* was 0.73 for CRC patients as compared with normal controls. No significant differences in stool *miR-221* levels were found between patients with proximal and distal CRCs. The use of antibiotics did not influence stool *miR-221* levels.

## 6. Hepatocellular Carcinoma

*MiR-221/222* is a critical modulator in the hepatocellular carcinoma (HCC) signaling pathway [22]. *MiR-221/222* was upregulated in the human liver in a fibrosis progression-dependent manner with upregulation of α1 (I) collagen (COL1A1) and α-smooth muscle actin (αSMA) [23,24,25]. Upregulation of *miR-221* and downregulation of p27Kip1 and p57Kip2 were associated with tumor stages, local recurrence, metastasis, and poor prognosis [24,25,26,27,28,29]. In a mouse model of liver cancer, *miR-221* overexpression stimulated growth of tumorigenic murine hepatic progenitor cells [30,31]. Inhibition of *miR-221* decreased liver cancer cell proliferation, clonogenicity, migration, and invasion and also induced G1 arrest and apoptosis *in vitro* and *in vivo* [22,27,32].

**Figure 1:**
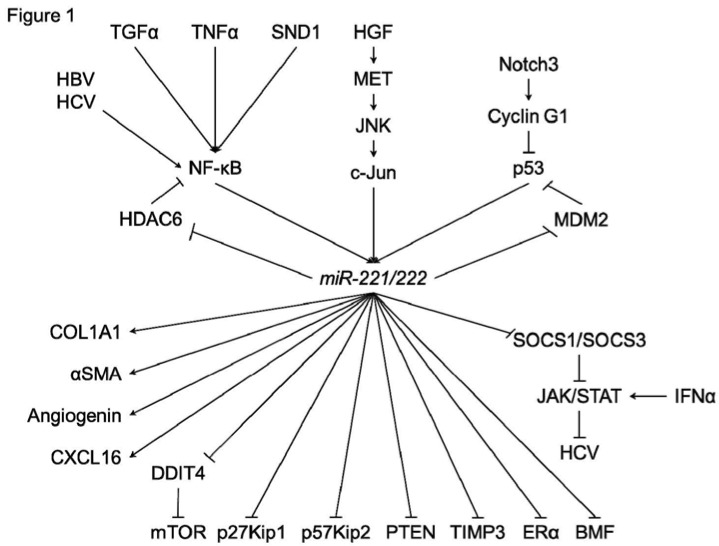
A schematic of the regulatory mechanisms of *miR-221/222* in hepatocarcinogenesis.

In HCC cells or hepatocyte, various functions of *miR-221/222* have been investigated (Figure 1). In addition to p27Kip1 and p57Kip2, several direct targets of *miR-221/222* were identified, such as estrogen receptor-alpha (ERα) and a proapoptotic BH3-only protein (BMF) [33,34]. DNA damage-inducible transcript 4 (DDIT4), a modulator of mTOR pathway, was also a direct target of *miR-221* [30]. Garofalo *et al.* showed that *miR-221/222*, by targeting PTEN and TIMP3 tumor suppressors, induce TRAIL resistance and enhance cellular migration through the activation of the AKT pathway and metallopeptidases. Xu *et al.* reported that *miR-221* was upregulated by HCV infection [35]. In addition, an *miR-221* mimic could accentuate the anti-HCV effect of IFN-α in an HCV model, through the inhibition of two members of the suppressor of cytokine signaling (SOCS) family, SOCS1 and SOCS3.

In HCC cells, regulation systems of *miR-221/222* have also been investigated (Figure 1). JNK/c-Jun activation and NF-κB nuclear translocation were reported to be essential for the transcription of *miR-221/222* [23,36,37]. Hepatitis B virus X protein (HBx) leads to the promotion of cell proliferation and cell growth viability with overexpression of *miR-221* [33]. HCV infection could also upregulate the expression of *miR-221* in an NF-κB dependent manner [35,38]. Staphylococcal nuclease domain-containing 1 (SND1) is a multifunctional protein that is overexpressed in multiple cancers, including hepatocellular carcinoma (HCC). Santheladur *et al.* reported that SND1-induced activation of NF-κB resulted in induction of *miR-221* and subsequent induction of angiogenic factors Angiogenin and CXCL16 [39].

Bae *et al.* showed that the direct suppression of HDAC6 (histone deacetylase 6) by *miR-221* was induced by JNK/c-Jun signaling in liver cancer cells but not in normal hepatic cells [36]. In addition, NF-κB could be activated by *miR-221*, since HDAC6 suppressed the translocation of NF-κB.

Fornari *et al.* reported that MDM2 (E3 ubiquitin-protein ligase homolog), a known p53 (TP53) modulator, is identified as a direct target of *miR-221* [40]. *MiR-221* can activate the p53/mdm2 axis by inhibiting MDM2 and, in turn, p53 activation contributes to *miR-221* enhanced expression. Giovannini *et al.* reported that Notch3 silencing in HCC resulted in p53 upregulation [41]. They found that Notch3 regulated p53 at post-transcriptional level controlling both Cyclin G1 expression and the feed-forward circuit involving p53, *miR-221*, and MDM2.

## 7. Pancreatic Cancer

Expression of *miR-221/222* is upregulated in pancreatic cancer as compared with normal pancreatic duct epithelial cells or normal pancreas tissues [42,43,44]. Pancreatic cancer patients with high *miR-221* expression had a relatively shorter survival compared to those with lower expression [42]. Antisense to *miR-221* suppressed the proliferative capacity, increased the amount of apoptosis, and sensitized the effects of gemcitabine in pancreatic cancer cells with concomitant up-regulation of PTEN, p27Kip1, p57Kip2, and PUMA, which are the tumor suppressors and the predicted targets of *miR-221* [42,45,46].

Tanaka *et al.* reported that metformin suppressed the expression of *miR-221* in human pancreatic cancer cells, leading to G1-phase arrest via the upregulation of p27Kip1 [47]. In addition, Sarker *et al.* reported that the treatment of pancreatic cancer cells with isoflavone mixture (G2535), formulated 3,3′-diindolylmethane (BR-DIM), or synthetic curcumin analogue (CDF) could downregulate the expression of *miR-221* and consequently upregulate the expression of PTEN, p27Kip1, p57Kip2, and PUMA, leading to the inhibition of proliferation and migration of pancreatic cancer cells [42]. Therefore, these agents combined with conventional chemotherapeutics could be useful in designing novel targeted therapeutic strategy for the treatment of pancreatic cancer.

Matrix metalloproteinases (MMPs) are closely related to cell migration and invasion. Among the MMPs, MMP-2 and MMP-9 have been implicated in human cancer invasion. Xu *et al.* reported that the tissue inhibitor of metalloproteinase (TIMP)-2 was directly regulated by *miR-221/222* [43]. They also showed that *miR-221/222* mimic directly inhibited TIMP-2 expression, leading to the upregulation of MMP-2 and MMP-9.

The platelet-derived growth factor (PDGF) signaling pathway has been found to play important roles in the development and progression of human cancers by regulating the processes of cell proliferation, apoptosis, migration, invasion, metastasis, and the acquisition of the epithelial-mesenchymal transition (EMT) phenotype. Su *et al.* reported that *miR-221* expression was activated by PDGF signaling [48]. After the inhibition of *miR-221*, PDGF did not alter the levels of cell migration, proliferation, and acquisition of the EMT phenotype. These results showed that *miR-221* is essential for the PDGF-mediated EMT phenotype, migration, and growth of pancreatic cancer cells. Downregulation of TRPS1 by *miR-221* is critical for PDGF-mediated acquisition of the EMT phenotype.

Plasma *miR-221* concentration could be a useful biomarker for cancer detection, monitoring tumor dynamics, and predicting malignant outcomes in pancreatic cancer patients [44]. Plasma *miR-221* levels were higher in pancreatic cancer patients than in benign pancreatic tumors and controls, and were correlated with distant metastasis. In addition, plasma *miR-221* levels were reduced in postoperative samples.

Pancreatic cysts are a group of lesions with heterogeneous malignant potential. *MiR-221* concentration in the endoscopically acquired pancreatic cyst fluid samples could be useful for the diagnosis of pancreatic cysts. *MiR-221* was expressed at higher levels in malignant cysts compared with benign or premalignant cysts [49].

## 8. Cholangiocarcinoma

In contrast to the other epithelial cancers, *miR-221/222* was downregulated in intrahepatic cholangiocarcinoma tissues, suggesting that *miR-221/222* would play onco-suppressive roles [25]. Okamoto *et al.* reported a relationship between *miR-221* expression and the sensitivity of cholangiocarcinoma (CCA) cells to gemcitabine [50]. Microarray analysis was used to determine the miRNA expression profiles of two CCA cell lines, HuCCT1 and HuH28. HuCCT1 cells were more sensitive to gemcitabine than were HuH28 cells, and 18 miRNAs were differentially expressed between HuH28 and HuCCT1. To determine the effect of candidate miRNAs on gemcitabine sensitivity, expression of each candidate miRNA was modified via either transfection of a miRNA mimic or transfection of an anti-oligonucleotide. Among these 18 miRNAs, ectopic overexpression of each of three downregulated miRNAs in HuH28 (*miR-29b*, *miR-205*, and *miR-221*) restored gemcitabine sensitivity to HuH28. Selective siRNA-mediated downregulation of either of two software-predicted targets, PIK3R1 (target of *miR-29b* and *miR-221*) or MMP-2 (target of *miR-29b*), also conferred gemcitabine sensitivity to HuH28.

## 9. Gastrointestinal Stromal Tumor (GIST)

Gastrointestinal stromal tumors (GISTs) are characterized by high expression of the KIT receptor tyrosine kinase protein, resulting from oncogenic mutations in the extracellular, juxtamembrane, or kinase domains. KIT is known to be directly regulated by *miR-221/222*, suggesting that *miR-221/222* would also play onco-suppressive roles in GISTs [51]. In fact, expression of *miR-221/222* is reduced in GISTs compared to control tissue and other sarcomas [51,52,53]. Overexpression of *miR-221/222* in GIST cells inhibited cell proliferation, affected cell cycle progression, and induced apoptosis [51,52].

Ihle *et al.* analyzed expression of *miR-221/222* in six KIT exon 9, three KIT exon 11 mutated, and nine wild-type GISTs [52]. MiRNA expression was lower for the wild-type compared to mutated GISTs. Transient transfection of *miR-221/222* reduced viability and induced apoptosis by inhibition of KIT expression and its phosphorylation and activation of caspases 3 and 7 in GIST cells. p-AKT, AKT, and BCL2 expression were also reduced after *miR-221/222* transfection.

## 10. Conclusions and Prospects

The discovery of the important role of miRNAs in cancer has opened up a new era of cancer investigations that take into account new and emerging knowledge regarding the RNA signaling systems. The unraveling of *miR-221/222* signaling pathways and networks will be key to understanding the role that deregulated miRNA functioning can play in oncogenic or onco-suppressive processes and may be important for defining novel therapeutic molecules.

Recently miRNAs contained in exosomes have been shown to be released and to act as a signal transducer. However, the function of secretory *miR-221/222* has never been reported. Previous reports showed that *miR-221/222* play various roles not only in cancer but also in vascular smooth muscle cells, vascular endothelial cells, and adipose tissue [54,55]. These suggest that interactions between cancers and blood vessels or adipose tissue would be mediated by secretory *miR-221/222*. Revealing the inter-organic functions of miRNAs will also help us to better understand cancer biology.
